# Simultaneous Determination of Ropivacaine and 3-Hydroxy Ropivacaine in Cerebrospinal Fluid by UPLC-MS/MS

**DOI:** 10.1155/2020/8844866

**Published:** 2020-12-04

**Authors:** Siyuan Chen, Jianshe Ma, Xianqin Wang, Quan Zhou

**Affiliations:** ^1^Institute of Forensic Science, Wenzhou Medical University, Wenzhou 325000, China; ^2^Analytical and Testing Center, School of Pharmaceutical Sciences, Wenzhou Medical University, Wenzhou 325035, China; ^3^The Laboratory of Clinical Pharmacy, The People's Hospital of Lishui, Lishui 323000, China

## Abstract

In this paper, a UPLC-MS/MS method was developed for the determination of ropivacaine and its metabolite 3-hydroxy ropivacaine in cerebrospinal fluid. The cerebrospinal fluid was processed by ethyl acetate liquid-liquid extraction. The multiple reaction monitoring (MRM) mode was used for quantitative analysis by monitoring the transitions of m/z 275.3 → 126.2 for ropivacaine, m/z 291.0 → 126.0 for 3-hydroxy ropivacaine, and m/z 290.2 → 198.2 for the internal standard. Standard curves for ropivacaine and 3-hydroxy ropivacaine in cerebrospinal fluid were conducted over the concentration range of 0.2–2000 ng/mL, demonstrating excellent linearity, and the lower limit of quantification was 0.2 ng/mL. The intraday precision of ropivacaine and 3-hydroxy ropivacaine was less than 11%, while the interday precision was less than 7%. The accuracy ranged between 87% and 107%, the average extraction efficiency was higher than 79%, and the matrix effect was between 89% and 98%. The developed method was then applied to a case of suspected poisoning of ropivacaine.

## 1. Introduction

Ropivacaine, which exists as the pure *S*-(–)-enantiomer, is a long-acting amide local anesthetic that can provide different effects at different doses [1]. At a concentration of 0.2%, it exhibits a stronger sensory nerve block and almost no motor nerve block, whereas at a concentration of 0.75%, it can the block motor nerve function [2, 3]. The fat solubility of this product is greater than lidocaine but less than bupivacaine, and its anesthetic strength is 8 times that of procaine [4, 5]. After epidural injection, the absorption is biphasic, and the fast and slow phase *t*_1/2_'s are 14 min and 4 h, respectively. During bilateral intercostal nerve blocks, its absorption into the blood is faster than with epidural injection. Ropivacaine is mainly metabolized in the liver, and its metabolites have a weak local anesthetic effect [6]. Clinically, it is mainly used for regional block anesthesia and epidural anesthesia [7, 8]. It is also used for regional block analgesia, such as analgesia after epidural surgery or labor [9]. Adverse reactions that can occur during epidural anesthesia are hypotension, bradycardia, nausea, and anxiety. When the blood concentration is too high, ropivacaine exhibits a dual effect by inhibiting and exciting the central nervous system, as well as inhibiting cardiac excitation, and prolonging the conduction of the cardiovascular system.

At present, various methods such as HPLC-UV [10], HPLC-DAD [11], LC-MS, or LC-MS/MS [12–20] have been developed for measuring the concentration of ropivacaine in biological fluids. The limits of quantitation of these methods ranged between 2.5 ng/ml and 75 ng/mL. However, to the best of our knowledge, none of these studies reported the quantitation of ropivacaine and its metabolite 3-hydroxy ropivacaine in cerebrospinal fluid by UPLC-MS/MS. In this paper, an UPLC-MS/MS method was developed for the determination of ropivacaine and its metabolite 3-hydroxy ropivacaine in cerebrospinal fluid, which was then applied to a case of suspected poisoning of ropivacaine.

## 2. Materials and Methods

### 2.1. Chemicals

**Figure 1:**
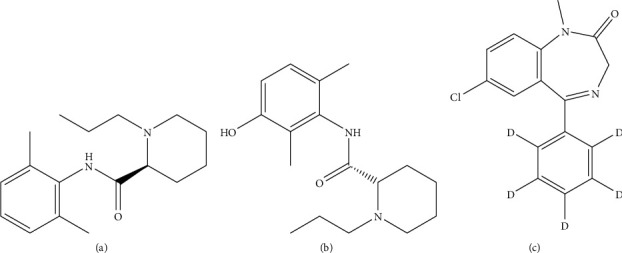
Chemical structures of ropivacaine (a), 3-hydroxy ropivacaine (b), and diazepam-d5 (c).

Ropivacaine, 3-hydroxy ropivacaine, and diazepam-d5 (internal standard), all with purities > 98% (Figure 1), were purchased from Sigma-Aldrich Co. LLC (St. Louis, MO, USA). Acetonitrile and methanol (HPLC grade) were purchased from Merck (Darmstadt, Germany). Ultrapure water was prepared by the Millipore Milli-Q purification system (Bedford, MA, USA).

### 2.2. Instruments and Methods

The UPLC-MS/MS was composed of an ACQUITY H-Class UPLC and XEVO TQS-micro triple quadrupole mass spectrometer (Waters Corp, Milford, MA, USA). A UPLC BEH C18 column (2.1 mm × 50 mm, 1.7 *μ*m, Waters, USA) was used as the stationary phase, and the column temperature was set at 40°C. The mobile phase consisted acetonitrile and 10 mmol/L ammonium acetate (containing 0.1% formic acid) in water. The mobile phase was gradient eluted with a flow rate of 0.4 mL/min. From 0 to 0.2 min, acetonitrile was 15% isocratic; from 0.2 min to 1.2 min, acetonitrile was increased from 15% to 85%; from 1.2 min to 2.1 min, acetonitrile was 85% isocratic; from 2.1 min to 2.4 min, acetonitrile was decreased from 85% to 15%; from 2.4 min to 4.0 min, acetonitrile was 15% isocratic.

**Figure 2:**
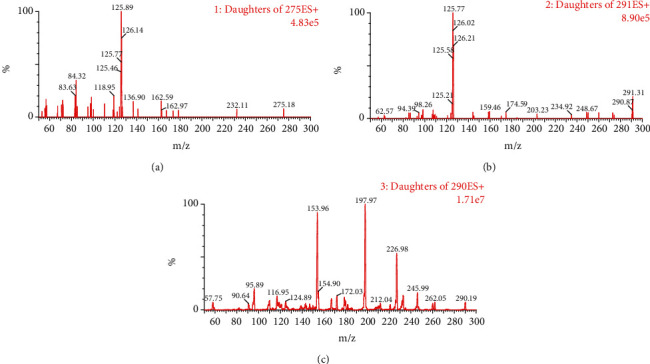
Mass spectra of ropivacaine (a), 3-hydroxy ropivacaine (b), and diazepam-d5 (c) in 10 mmol/L ammonium acetate (containing 0.1% formic acid) in water and acetonitrile (1 : 1, *v*/*v*).

The desolvation gas (nitrogen) was set to 850 L/h, and the cone gas was set to 50 L/h. The capillary voltage was set to 1.5 kV, and the desolvation temperature was 450°C. The MRM mode was used for quantitative analysis. The selected MRM transitions were as follows: m/z 275.3 → 126.2 (quantification) and m/z 275.3 → 84.3 (confirmation) for ropivacaine (cone voltage 30 V, collision voltage 20 V); m/z 291.0 → 126.0 (quantification) and 291.0 → 98.3 (confirmation) for 3-hydroxy ropivacaine (cone voltage 30 V, collision voltage 20 V); m/z 290.2 → 198.2 (quantification) and 290.2 → 154.0 (confirmation) for diazepam-d5 (cone voltage 25 V, collision voltage 30 V) (Figure 2**)**.

### 2.3. Preparation of Reference Solutions

Stock solutions of ropivacaine (1.0 mg/mL), 3-hydroxy ropivacaine (0.1 mg/mL), and diazepam-d5 (0.1 mg/mL) were prepared in methanol. The stock solutions were diluted with methanol to prepare a series of standard working solutions, and all solutions were stored at 4°C until use.

### 2.4. Standard Curve Preparation

Blank cerebrospinal fluid was mixed with an appropriate amount of the standard working solutions to prepare a curve for ropivacaine and 3-hydroxy ropivacaine with concentrations of 0.2, 1, 5, 20, 100, 500, 1000, and 2000 ng/mL in cerebrospinal fluid. Quality control (QC) samples (0.5, 90, and 1500 ng/mL) were prepared in the same manner as the standard curve samples. The standard curve samples were treated by ethyl acetate liquid-liquid extraction and analyzed by UPLC-MS/MS.

### 2.5. Sample Processing

Cerebrospinal fluid (100 *μ*L), the internal standard solution (10 *μ*L of a 0.5 *μ*g/mL solution), and ethyl acetate (2 mL) were added to a 5 mL centrifugation tube, which was then vortexed for 1.0 min. After centrifugation (3000 rpm, 4°C, 10 min), the supernatant (1.5 mL) was removed and dried under a stream of air and then reconstituted in 100 *μ*L of methanol. The resulting solution (80 *μ*L) was transferred into the inner liner of the injection bottle, 2 *μ*L of which was injected into the UPLC-MS/MS for analysis.

### 2.6. Method Validation

The validation method was established in accordance with the US Food and Drug Administration Bioanalysis Method Validation Guidelines [21]. The selectivity of the method was evaluated by analyzing blank cerebrospinal fluid, and the blank cerebrospinal fluid spiked with ropivacaine, 3-hydroxy ropivacaine, and the internal standard by UPLC-MS/MS. For the standard curves, the standard solutions (0.2, 1, 5, 20, 100, 500, 1000, and 2000 ng/mL) of ropivacaine and 3-hydroxy ropivacaine were prepared in cerebrospinal fluid. Under the same conditions, the area under each peak was measured, a standard curve was drawn with the peak area plotted against the concentration, and the linearity of the standard curve was evaluated. The lower limit of quantitation (LLOQ) was defined as the lowest concentration that could be detected with a signal-to-noise ratio (S/N) exceeding 10. The precision and accuracy of the LLOQ should be less than 20% and within 80%-120%, respectively. The detection limit of the signal-to-noise ratio was 3.

The precision and accuracy were evaluated by measuring QC samples at three separate concentrations in six replicates. Precision is expressed as a relative standard deviation (RSD), and the intraday and interday precisions were determined by measuring QC samples at three concentration levels on three consecutive days. The intraday and interday accuracies were reported based on the average value of the QC samples at the three different concentrations and the true value for the three consecutive days. The extraction efficiency was evaluated by comparing the measured peak area of the low-, medium-, and high-concentration QC samples to the corresponding standard peak area. The matrix effect was evaluated by comparing the peak area of the low-, medium-, and high-concentration standard solution in the blank cerebrospinal fluid after sample treatment to the peak area of the corresponding standard solution.

To confirm the ability to measure the diluted samples from beyond the upper limit of quantitation to within the calibration concentration range [22], six aliquots of ropivacaine (20,000 ng/mL) were diluted 20-fold with blank cerebrospinal fluid for analysis. The peak area of the standard (20,000 ng/mL in cerebrospinal fluid) after the 20-fold dilution was compared to that of 1000 ng/mL undiluted sample. The acceptance criteria for ropivacaine consisted a precision (RSD) ≤ 15% and an accuracy of 85-115% after all six determinations [23].

The stability of ropivacaine and 3-hydroxy ropivacaine in cerebrospinal fluid was investigated by analyzing the QC samples of three low-, medium-, and high-concentration levels placed under three storage conditions, which include long-term stability (-20°C, 30 days), short-term stability (2 h at room temperature), and freeze-thaw stability (3 consecutive freezing and thawing cycles for 3 days) (-20°C to room temperature).

## 3. Result and Discussion

### 3.1. Method Development

The choice of positive and negative mode for ESI is often evaluated in methodology [24–26]. The experiment found that the ESI positive ion mode was more sensitive than the negative ion mode. After optimization of the mass spectrometry conditions, the appropriate cone voltage and collision voltage were selected as the mass spectrometry detection parameters, and the fragment peaks with high relative intensities were selected as the quantitative ion pairs.

The liquid chromatography conditions should be chosen in order to effectively separate endogenous interfering substances [27–29] from ropivacaine, 3-hydroxy ropivacaine, and internal standard. During the method development, a series of mobile phases were examined and compared: methanol or acetonitrile (containing 0.1% formic acid), 10 mmol/L ammonium acetate buffer solution (containing 0.1% formic acid) in acetonitrile or methanol, and 10 mmol/L ammonium acetate buffer solution (containing 0.05% ammonia) in methanol or acetonitrile. Under comprehensive comparison, the gradient elution effect of 10 mmol/L ammonium acetate buffer solution (containing 0.1% formic acid) in acetonitrile afforded the best results, such that the peak shapes of ropivacaine and 3-hydroxy ropivacaine were narrow and the retention times were adjectives.

### 3.2. Method Validation

**Figure 3:**
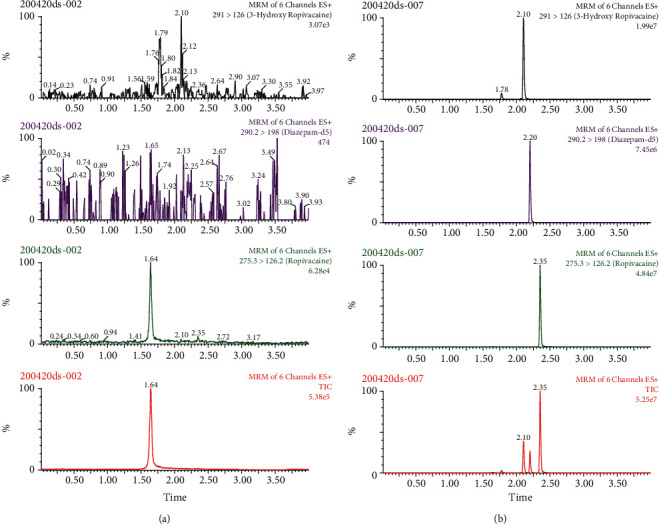
UPLC-MS/MS chromatograms of ropivacaine, 3-hydroxy ropivacaine, and diazepam-d5 in cerebrospinal fluid. (a) Blank cerebrospinal fluid. (b) Blank cerebrospinal fluid spiked with ropivacaine, 3-hydroxy ropivacaine, and diazepam-d5.

The typical UPLC-MS/MS chromatograms of blank cerebrospinal fluid, fortified cerebrospinal fluid with ropivacaine, 3-hydroxy ropivacaine, and the internal standard are shown in Figure 3. The retention times of ropivacaine, 3-hydroxy ropivacaine, and the internal standard were 2.35 min, 2.10 min, and 2.20 min, respectively. No obvious coextractives interfered with the detection.

The standard curves of ropivacaine and 3-hydroxy ropivacaine in cerebrospinal fluid were acquired over the concentration range of 0.2–2000 ng/mL. The equations for the standard curve of ropivacaine and 3-hydroxy ropivacaine were *y* = 0.4069*x* + 0.2476, *r* = 0.9998, and *y* = 0.5768*x* + 0.3461, *r* = 0.9992, where *y* represents the ratio of the peak area of ropivacaine and 3-hydroxy ropivacaine to the internal standard and *x* represents the concentration of ropivacaine and 3-hydroxy ropivacaine in cerebrospinal fluid. The LLOQ of ropivacaine and 3-hydroxy ropivacaine in cerebrospinal fluid was determined to be 0.2 ng/mL. The detection limit of ropivacaine and 3-hydroxy ropivacaine in cerebrospinal fluid was 0.05 ng/mL, and the signal-to-noise ratio was 3.

It can be seen from Table 1 that the intraday precision of ropivacaine and 3-hydroxy ropivacaine was less than 11%, the interday precision was less than 7%, and the accuracy range was between 87% and 107%. In addition, the average extraction efficiency was higher than 79%, and the matrix effect was between 89% and 98%. It was shown that the established UPLC-MS/MS method met the requirements for the analysis of biological samples of ropivacaine and 3-hydroxy ropivacaine with respect to precision, accuracy, extraction efficiency, and matrix effect.

After the 20-fold dilution of the six aliquots of ropivacaine (20,000 ng/mL) in cerebrospinal fluid, the accuracy (*n* = 6) was identified as 98.5%, and the precision (RSD, *n* = 6) was 5.7%. These results met the acceptance criteria, suggesting that samples exceeding the calibration curve concentrations can be diluted 20-fold to achieve concentrations within the range of the assay.

After analysis of the three stability conditions, which consisted of room temperature for 2 hours, –20° C for 30 days, and 3 freezing and thawing cycles, the accuracy of ropivacaine and 3-hydroxy ropivacaine in cerebrospinal fluid was between 85% and 109%, and the RSD was within 12%. These results indicate that ropivacaine and 3-hydroxy ropivacaine were both stable.

### 3.3. Application

A woman with pain in the right elbow was diagnosed with tennis elbow and cervical spondylosis after a physical examination, cervical X-ray, and cervical magnetic resonance imaging. The patient was then given local injections, which consisted 20 mg of ropivacaine mesylate, 20 mg of sodium hyaluronate, 3.0 g of adenosine cobaltamine, and 10 mg of tramalin dissolved in 10 mL of saline, in the facet joints of the cervical spine to improve elbow pain. During injection therapy, the patient experienced chest tightness and difficulty breathing. The treating physician immediately stopped the injection and gave oxygen inhalation, mouth-to-mouth artificial respiration, injection of respiratory stimulants, and continuous-breathing mask pressure to maintain respiration. After extracorporeal cardiac compressions, an injection of 1 mg norepinephrine, continuous cardiac compression, balloon breathing, and other treatments, rescue continued for about 40 minutes. At last, the patient's breathing and heartbeat showed no signs of recovery, and the rescue was declared invalid. The disputes over the cause of death requested ropivacaine testing of the cerebrospinal fluid. The concentrations of ropivacaine and 3-hydroxy ropivacaine in the cerebrospinal fluid were 22.8 *μ*g/mL and 0.57 ng/mL by the developed UPLC-MS/MS, respectively. According to the literature, a concentration of 2.2 *μ*g/mL of ropivacaine in plasma was reported to be toxic [30], whereas the toxic concentration in cerebrospinal fluid was unknown.

## 4. Conclusion

In this study, a sensitive, rapid, and selective UPLC-MS/MS method was established for the determination of ropivacaine and 3-hydroxy ropivacaine in cerebrospinal fluid with a linear range of 0.2–2000 ng/mL. UPLC-MS/MS has a faster analysis time and higher sensitivity than traditional HPLC. It only takes 4 min to complete the analysis of one cerebrospinal fluid sample, which could save a significant amount of time and resources. It was also successfully applied to a ropivacaine poisoning case.

## Figures and Tables

**Table 1:** Precision, accuracy, extraction efficiency, and matrix effect data of ropivacaine and 3-hydroxy ropivacaine in cerebrospinal fluid *n* = 6.

Compound	Concentration (ng/mL)	Accuracy (%)		Precision RSD (%)		Matrix effect	Extraction efficiency
		Intraday	Interday	Intraday	Interday		
Ropivacaine	0.2	91.9	93.5	10.0	6.6	97.1	80.2
	0.5	93.2	99.2	6.8	5.9	94.9	84.1
	90	106.2	106.6	2.7	3.4	96.3	87.9
	1500	101.2	100.4	3.1	3.8	93.4	87.2

3-Hydroxy ropivacaine	0.2	88.7	87.1	7.7	3.3	90.9	79.9
	0.5	105.6	96.1	4.3	5.5	93.9	83.3
	90	95.9	103.4	5.3	1.8	92.5	85.0
	1500	102.2	97.1	4.3	5.5	89.8	84.3

## Data Availability

The data used to support the findings of this study are included within the article.
